# Supplementary material for: Reversion analysis reveals the in vivo immunogenicity of a poorly MHC I-binding cancer neoepitope
Source: Nat Commun. 2021 Nov 5;12:6423. doi: 10.1038/s41467-021-26646-5 (PMC8571378; doi:10.1038/s41467-021-26646-5)
Supplement: Supplementary file 1 — Supplementary Information [file 41467_2021_26646_MOESM1_ESM.pdf]

Supplementary Information

Revised title: Reversion analysis reveals the in vivo immunogenicity of a poorly MHC I-binding cancer neoepitope

Ebrahimi-Nik et al.

Supplementary Figures 1-9

Supplementary Tables 1-5

Supplementary References

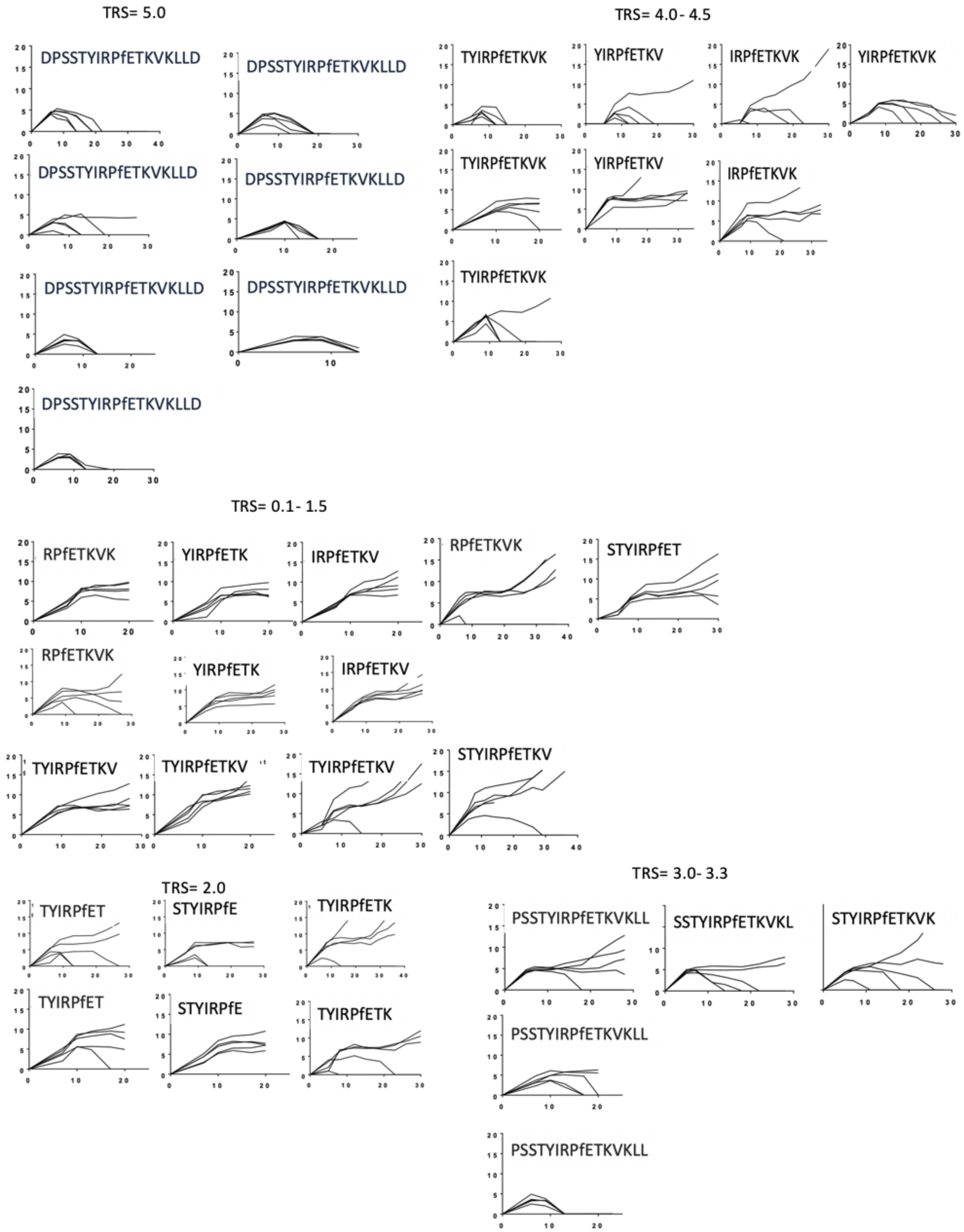

Supplementary Fig. 1. Data on tumor rejection elicited by individual peptides shown in groups in Fig. 1c. Several truncated versions of the 18-mer *Ccdc85c*^{MUT} peptide (as indicated in each panel) were tested in tumor rejection assay as in Fig. 1c. BALB/cJ mice were immunized and tumor challenged. Each line represents tumor growth in a single mouse. Tumor rejection score (TRS) for each group of neoepitopes is shown, where five represents a complete tumor protection and zero means no tumor rejection. The raw data for each panel are provided as raw data Excel file for Supplementary Fig. 1.

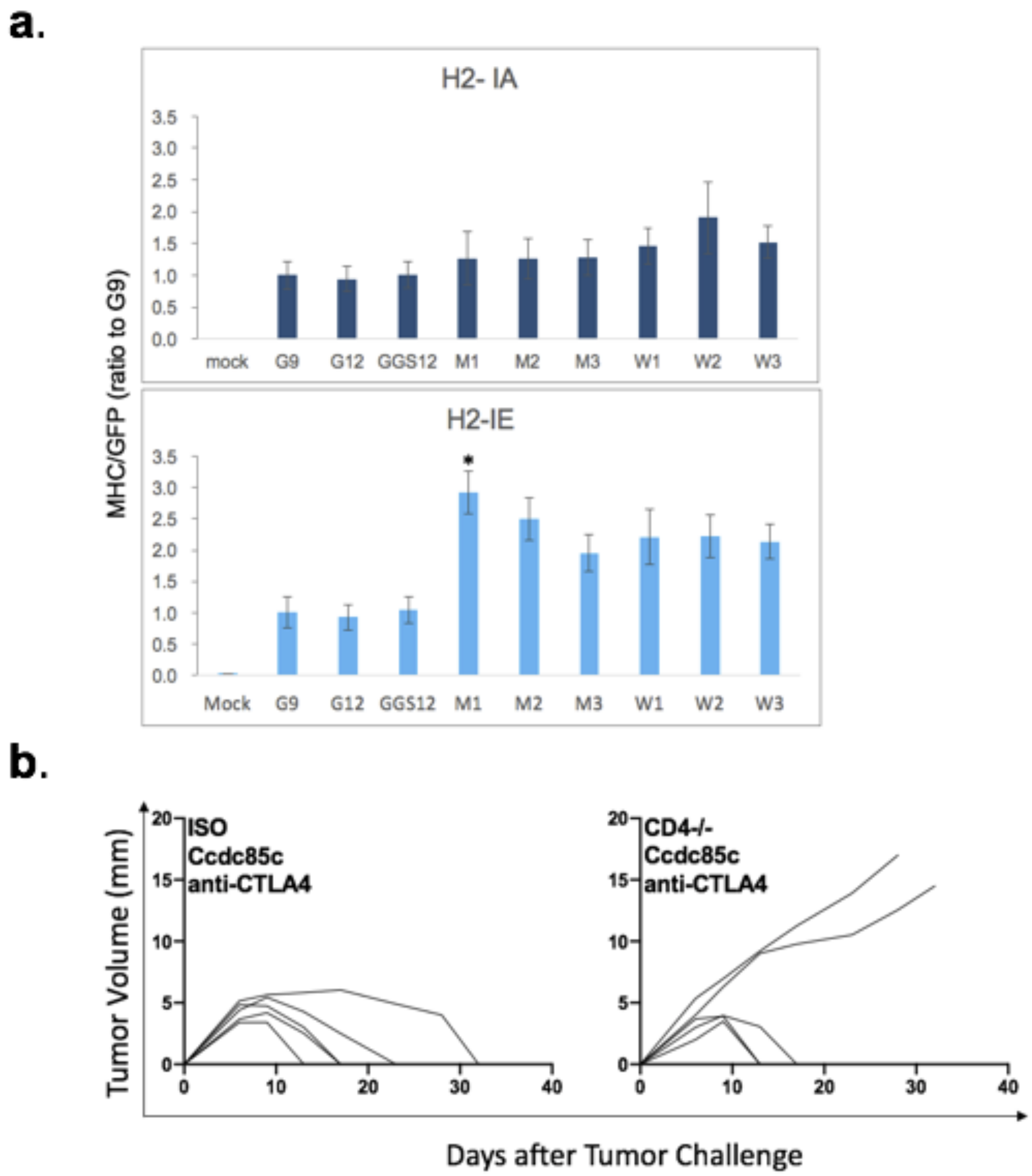

Supplementary Fig. 2. Assessing CD4 response to Ccdc85c^{MUT}. a Presentation of *Ccdc85c^{MUT}* and *Ccdc85c^{WT}* peptides by H2-IA and H2-IE are measured by cell-surface density assay. The y-axis indicates MHC/GFP (ratio of MHC MFI to GFP MFI) for MHC II-peptide, normalized to the MHC/GFP of the negative control peptide, G9, 9-mer all-glycine. Four technical and two independent biological repeats of this experiment have been practiced. The following additional negative control peptides are used: G12 (12-mer all-glycine) and GGS12 (4x(glycine-glycine-serine). **b.** Naïve BALB/cJ mice were injected with 250 µg of CD4 depleting GK1.5 antibody or isotype control during the priming phase (6 days before the first immunization). Mice were immunized, as described in the Methods, with Ccdc85cMUT and were challenged with 95,000 Meth A cells. The differences between tumor growth curves between un-depleted and CD4-depleted mice are not statistically significant.

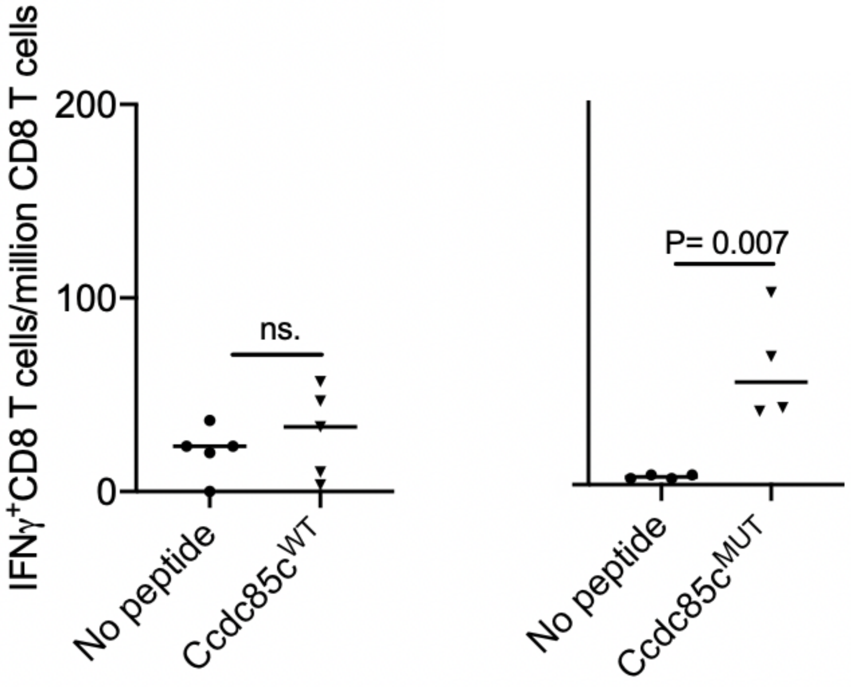

Supplementary Fig. 3. CD8 response to Ccdc85c^{MUT} and Ccdc85c^{WT} peptides. BALB/c mice were immunized twice at a weekly interval with 18-mer peptides containing Ccdc85c^{WT} (left) or Ccdc85c^{MUT} (right) as described in Methods. Spleens were harvested seven days after the second immunization and CD8 T cells stimulated in vitro with the peptides (10 µM concentration) used for immunization, as indicated. CD8 cells were analyzed for release of interferon γ. Each dot represents a single mouse. The P values were calculated using two tailed t-test. Data are presented as mean values +/- SD.

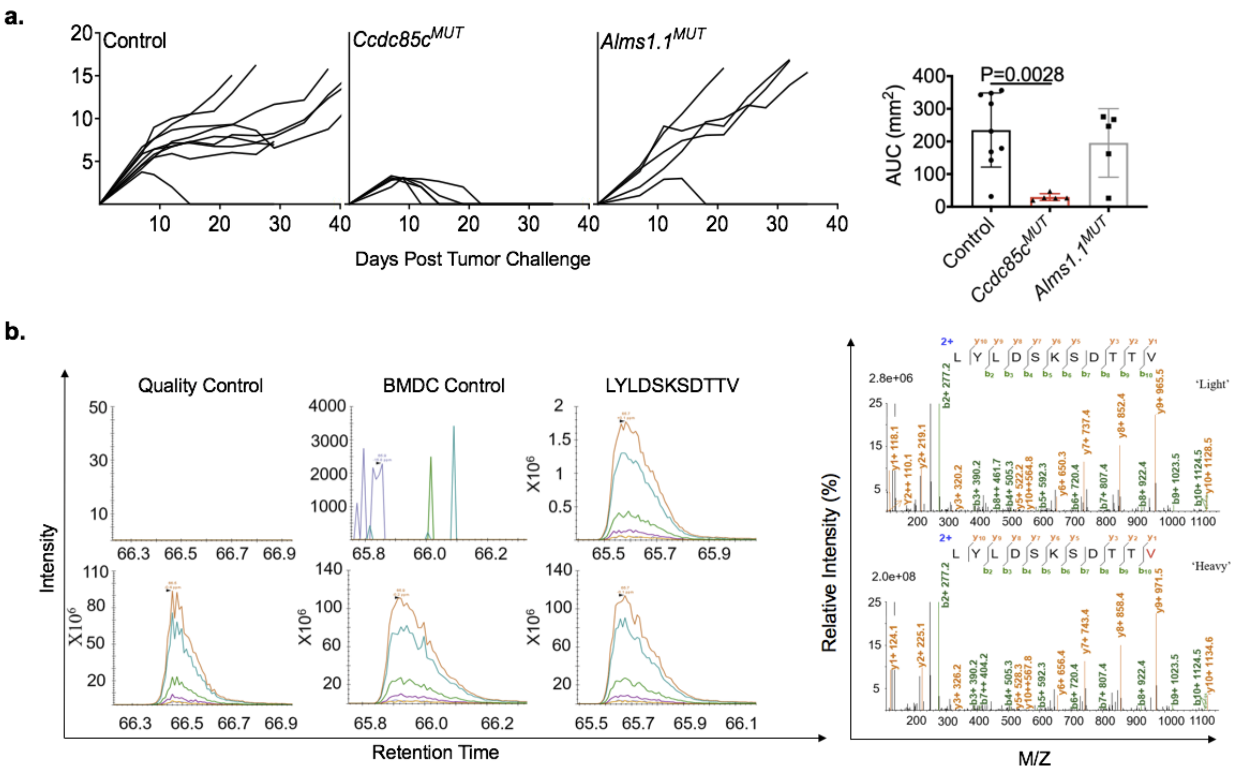

Supplementary Fig. 4. Tumor rejection efficacy of the control peptide, *Alms1.1*^{MUT} and its targeted MS-based detection. a. BALB/cJ mice were immunized with DMSO (as control), *Alms1.1*^{MUT} or *Ccdc85c*^{MUT} twice and were tumor challenged with Meth A cells as described in Methods. Each line represents tumor growth in a single mouse (control group; n=9, Ccdc85c^{MUT}; n= 5, and Alms1.1^{MUT}; n= 5). AUC scores for tumor growth curves are shown at the right panel. Data are presented as mean values +/- SD. The P values for AUC score comparisons were calculated using the 1-way ANOVA test and then were adjusted using the Dunnett's multiple comparison test. **b**. Targeted MS-based detection of the MHCI neoepitope LYLDSKSDTTV is shown. Heavy labeled synthetic peptides were spiked into the peptide samples; the mutation is in red. The matched peak lists for the "heavy" and "light" ions were extracted and monitored, while only single charge y ions were plotted. First, the absence of "light" peptide and the presence of the "heavy" peptide were confirmed by PRM as a quality control measure in the synthetic peptide samples (upper left and lower left, respectively). Then, the co-elution of the synthetic "heavy" and endogenous "light" fragment ions was measured by PRM in *Alms1.1*^{MUT} pulsed BMDC MHCI peptide sample. Peptides eluted from un-loaded BMDCs were measured as a negative control. Representative resulting MS/MS spectra of the "light" and "heavy" counterparts are provided. Figures were edited to improve resolution and readability.

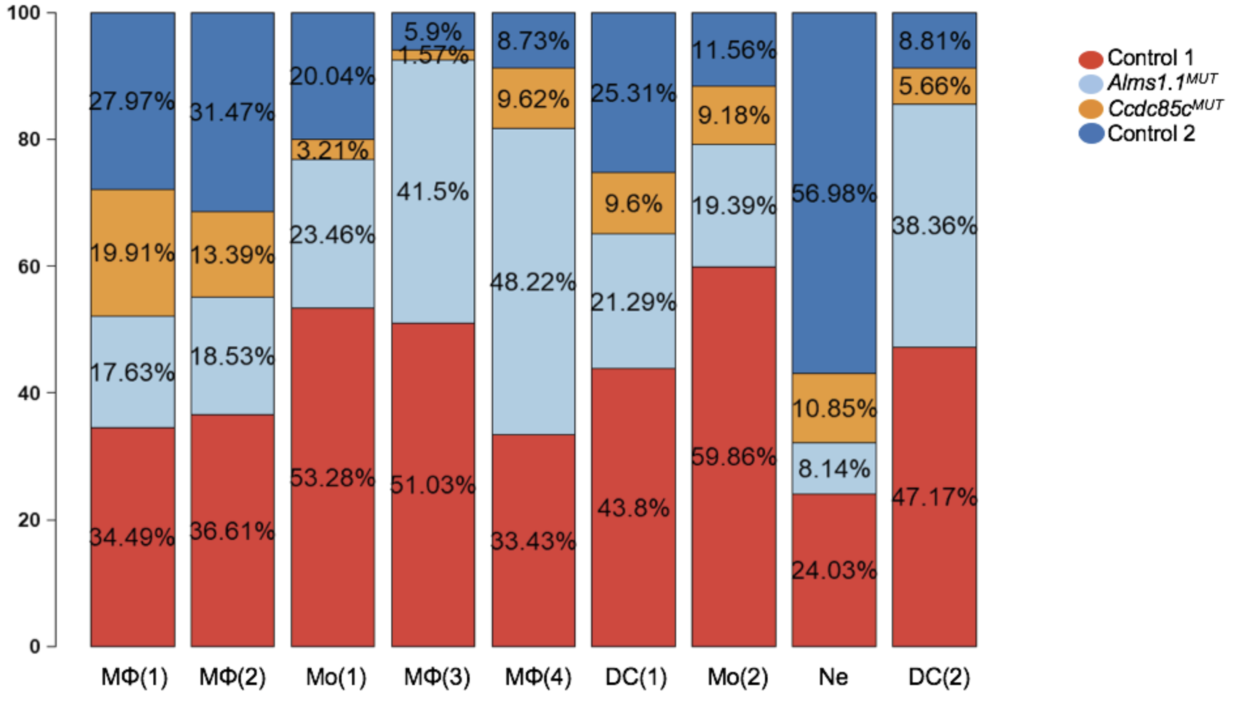

Supplementary Fig. 5. The library composition plot for myeloid cell clusters in mice immunized with *Ccdc85c*^{MUT}, *Alms1.1*^{MUT} and BMDCs. Tumors from mice immunized with *Ccdc85c*^{MUT}, *Alms1.1*^{MUT} and BMDCs (control group) were harvested ten days after the tumor challenge. Tumor infiltrating CD45+ cells were sorted and analyzed by scRNA sequencing as described in Methods. Combined scRNA sequencing data from four libraries were analyzed. The library composition plot for each myeloid cell type is shown. Please refer to page 5, 6 and 12 of the paper for definition of each cell type.

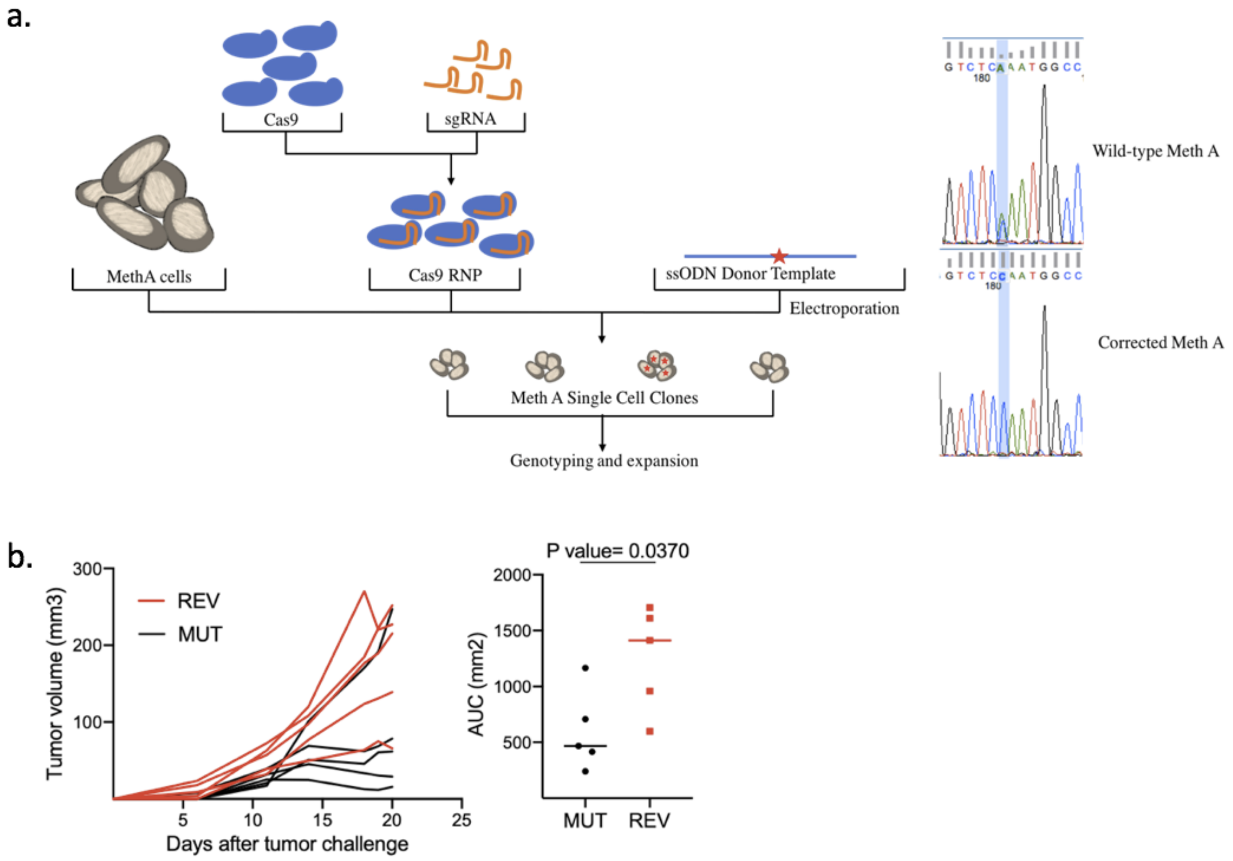

Supplementary Fig. 6. Process of CRISPR editing the Meth A cell line and its effect on kinetics of tumor growth a. Schematic diagram of CRISPR mediated reversion of point mutation in *Ccdc85c*^{MUT} Meth A tumor cells is shown. Sequence of the wild type (MUT1) and corrected Meth A (REV) at the genomic position of chr12-108221754 are shown at the right panel. **b**. Naïve BALB/cJ mice were challenged with REV or MUT cell line. Each line represents tumor growth in a single mouse. On the right panel, total Area Under the Curve (AUC) scores for REV and MUT are plotted. Each bar shows the average total AUC score for the indicated group. Error bars represent standard deviation (SD). *P* values were calculated using two tailed t-test (n= 5 per group).

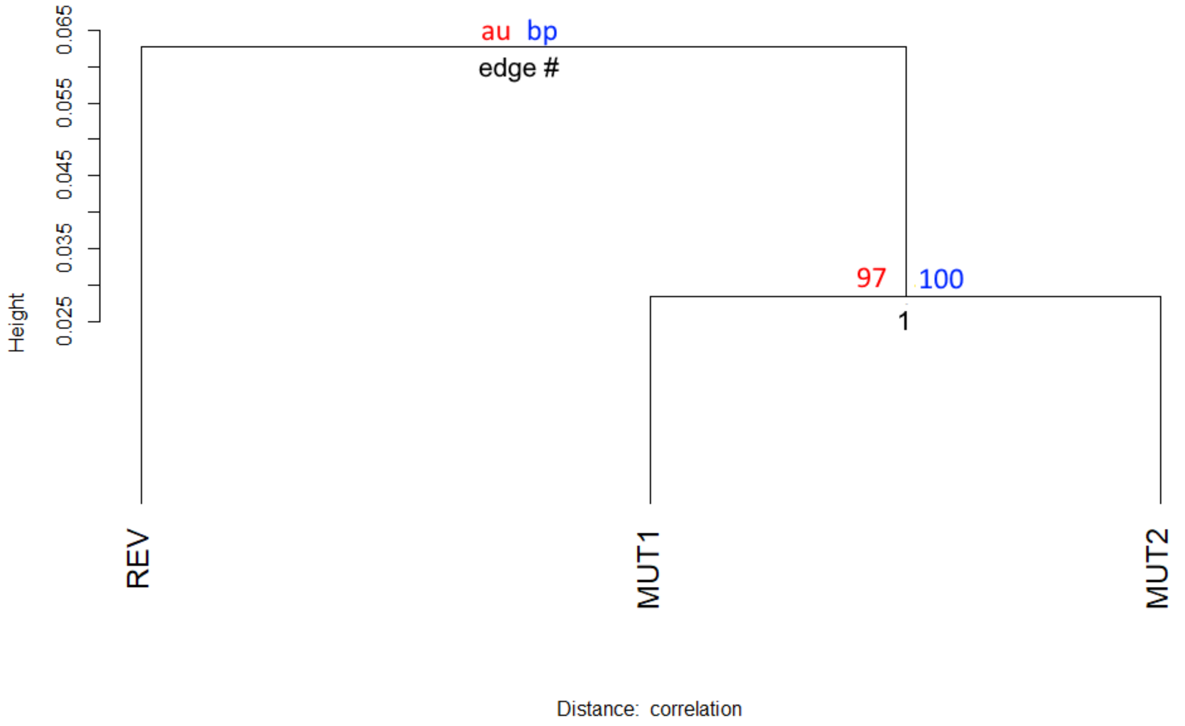

Supplementary Fig 7. The plot shows the Approximately Unbiased (AU, in red), p-value and the Bootstrap Probability (BP, in Green) value for the edge (to MUT1 and MUT2 sub-tree) using pvclust R package [1] for assessing the uncertainty in hierarchical cluster analysis and calculated via bootstrap resampling. Values on the edges of the clustering are p-values given in % such as clusters with AU larger than 95% are considered strongly supported by the data.

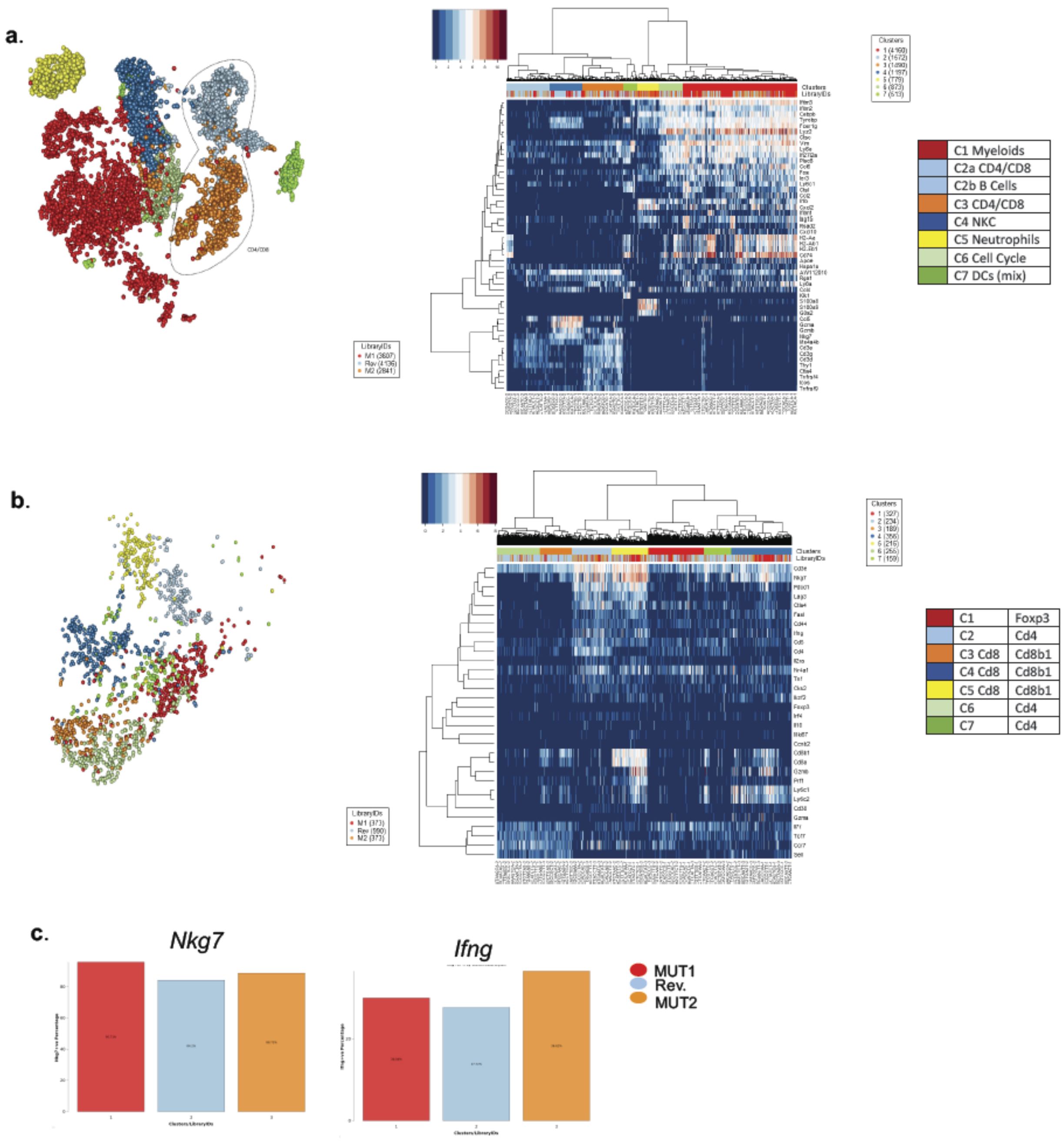

Supplementary Fig. 8. a. The process of analyzing TME of MUT1, REV and MUT2. Tumors from mice challenged with MUT1, REV and MUT2 Meth A cell lines were harvested ten days after the tumor challenge. Tumor infiltrating CD45+ cells were sorted and analyzed by scRNA sequencing as described in Methods. Combined scRNA sequencing data from the three libraries (MUT1, REV and MUT2) were classified into 7 cell clusters. **b**. Two main *Cd4* and *Cd8* T cell clusters were analyzed by further sub-clustering. Pure CD8 T cells (C3, C4 and C5) were computationally extracted for further analysis. **c**. Percentage of *Nkg7* and *Ifng* gene expression in CD8 T cells of MUT1, MUT2 and REV libraries are shown.

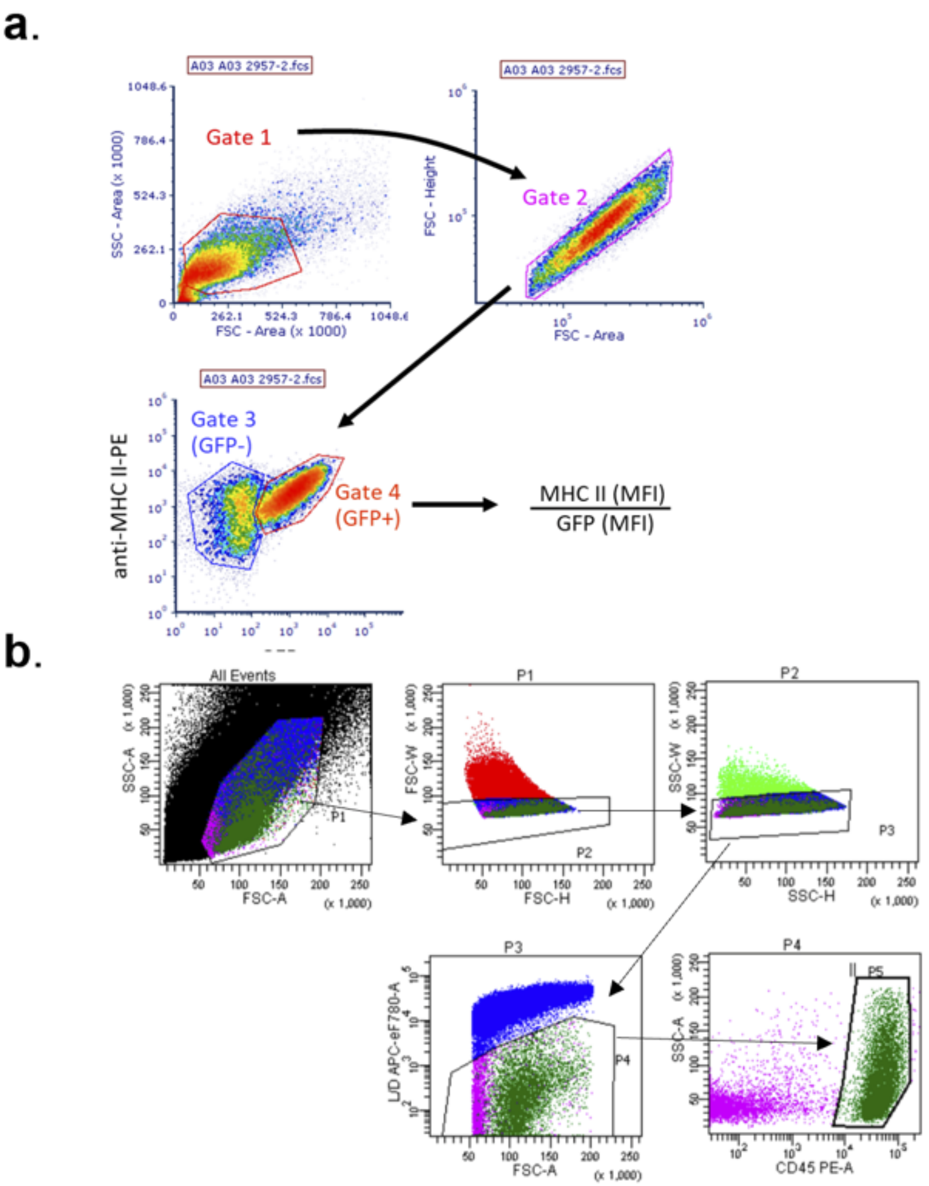

Supplementary Fig. 9. Gating strategy. a. Shows gating strategy for MHC II binding analysis. NIH3T3 cells that stably expressed MHC II alpha chain and transiently expressed MHC II beta chain were gated based on the forward scatter (FSC) and side scatter (SSC) properties (Gate 1). Singlets gates (Gate 2) were made based on the height and area of the FSC. MHC II expression levels in GFP-positive cells (Gate 4) were determined by calculating the ratio of MHC II (MFI) to GFP (MFI). **b.** Represents gating strategy for sorting CD45 positive cells of Meth A TME. Lymphocytes were gated based on the FSC-A and SSC-A (P1). Cells were further gated for singlets (P2 and P3). Single cells that were negative for eflour780 live/dead stain were gated as alive cells in P4. Live single cells that were positive for CD45-PE were sorted as shown in the gate P5.

Supplementary Table 1. The list of peptides and nucleotide sequences used for cell-surface MHC density assay.

Peptide ID	Original* length	Amino acid sequence used for assay	Nucleotide sequence
M1	11mer	** GTYIRPFETKVK	*** ggg act tac atc agg cca ttt gag acc aag gtg aag
M2	10mer	GGYIRPFETKVK	ggg ggc tac atc agg cca ttt gag acc aag gtg aag
M3	9mer	GGGIRPFETKVK	ggg ggc gga atc agg cca ttt gag acc aag gtg aag
W1	11mer	GTYIRPLETKVK	ggg act tac atc agg cca ttg gag acc aag gtg aag
W2	10mer	GGYIRPLETKVK	ggg ggc tac atc agg cca ttg gag acc aag gtg aag
W3	9mer	GGGIRPLETKVK	ggg ggc gga atc agg cca ttg gag acc aag gtg aag

*Original length represents length of the peptide without additional glycine.
**A complementary all-glycine amino acid sequence is added to the N terminus of each peptide to adjust its length to 12-mer (to eliminate length-dependent effects of the peptides).
*** Complementary all-glycine codon nucleotide sequences are added to the N terminus of each DNA construct to adjust the length of the corresponding peptide to 12-mer.

Supplementary Table 2. Enriched CDR3 GLIPH motifs of TCRs from TILs of MUT1 library. P values were calculated using GLIPH package calculation.

Motif	Counts	avgRef	topRef	OvE	p-value
YA	32	0.72	4	44.137	0.001
YAE	32	0.07	2	438.356	0.001
QDT	26	0.68	5	37.956	0.001
AET	20	1.05	6	18.939	0.001
NER	20	0.01	1	1111.111	0.001
QNT	18	0.77	6	23.225	0.001
DW	18	1.49	7	12.008	0.001
TGQ	18	1.46	6	12.244	0.001
DWG	15	0.38	4	39.164	0.001
NTGQ	14	0.01	1	736.842	0.001
NQA	13	0.03	2	333.333	0.001
NYAE	13	0	1	1444.444	0.001
NYA	13	0.02	1	590.909	0.001
NSD	12	0.07	2	171.428	0.001
SNER	11	0	1	3666.666	0.001
SAET	10	0.05	2	178.571	0.001
SAE	10	0.39	4	25.062	0.001
GQD	9	0.59	4	15.075	0.001
NQD	9	0.03	1	272.727	0.001
NQDT	9	0.01	1	692.307	0.001
ANSD	8	0	1	1142.857	0.001
ANS	8	0.41	3	19.23	0.001
DWGG	8	0.06	2	133.333	0.001
GNYA	8	0.01	1	799.999	0.001
GQDT	8	0.14	2	54.421	0.001
SQN	7	0.34	3	20.527	0.001
TGGA	7	0.44	5	15.59	0.001
GDA	7	0.26	2	26.923	0.001
SQNT	7	0.04	1	159.09	0.001
NNQA	6	0	1	3000	0.001
GQNT	6	0.27	3	21.582	0.001
NNQ	6	0.33	3	18.126	0.001
RLGG	6	0.21	3	27.397	0.001
GLGG	6	0.35	4	16.806	0.001
SDA	6	0.32	3	18.461	0.001
GGQD	5	0.1	2	49.999	0.001
LGGR	5	0.34	3	14.577	0.001
GGAR	5	0.2	3	24.999	0.001
DWGA	4	0.03	1	121.212	0.001
DAR	4	0.19	3	20.304	0.001
YYA	4	0	1	1000	0.001
RDWG	4	0.11	3	36.036	0.001
GSQN	4	0.02	1	173.913	0.001
LYAE	3	0	1	1000	0.001
GYAE	3	0.01	1	166.666	0.001
DQDT	3	0	1	600	0.001
WGNY	3	0.02	1	124.999	0.001
ISNE	3	0.01	1	249.999	0.001
RYAE	3	0	1	750	0.001

Supplementary Table 3. Enriched CDR3 GLIPH motifs of TCRs from TILs of REV library. P values were calculated using GLIPH package calculation.

Motif	Counts	avgRef	topRef	OvE	p-value
AE	145	14.25	31	10.171	0.001
YAE	61	0.16	3	376.543	0.001
YA	61	1.65	6	36.858	0.001
AET	57	2.45	7	23.246	0.001
QN	57	5.38	14	10.586	0.001
QDT	50	1.65	7	30.193	0.001
QNT	49	1.91	8	25.641	0.001
NER	47	0.04	2	999.999	0.001
TGQ	45	3.58	12	12.562	0.001
DW	44	3.71	13	11.856	0.001
DWG	35	0.98	8	35.46	0.001
NTGQ	32	0.04	2	711.111	0.001
SNER	32	0.01	1	1684.21	0.001
SAE	31	1.02	5	30.214	0.001
SAET	31	0.17	3	177.142	0.001
NYA	29	0.05	2	557.692	0.001
NYAE	29	0.01	1	1705.882	0.001
SQNT	25	0.09	2	268.817	0.001
SQN	25	0.74	6	33.692	0.001
GDA	25	0.6	5	41.05	0.001
NQA	20	0.08	2	246.913	0.001
NQDT	16	0.02	1	666.666	0.001
NQD	16	0.08	3	188.235	0.001
NSD	16	0.22	3	71.748	0.001
ISNE	13	0.04	1	288.888	0.001
ISN	13	0.39	5	33.333	0.001
TGGA	13	1.18	5	10.924	0.001
SDA	11	0.77	5	14.248	0.001
NNQ	11	0.85	5	12.82	0.001
NNQA	10	0	1	1111.111	0.001
NSGN	10	0.46	3	21.739	0.001
GQNT	10	0.68	4	14.513	0.001
GNYA	10	0.02	1	434.782	0.001
GLGG	9	0.86	6	10.428	0.001
GQDT	9	0.36	3	24.523	0.001
TSAE	9	0.05	2	155.172	0.001
WGY	8	0.69	5	11.577	0.001
DWGG	8	0.12	4	66.666	0.001
ASA	8	0.67	5	11.94	0.001
RDWG	8	0.29	3	27.586	0.001
ASAE	8	0.03	1	216.216	0.001
GSQN	7	0.05	2	127.272	0.001
RLGG	7	0.57	4	12.089	0.001
DWGY	7	0.01	1	636.363	0.001
GYA	7	0.23	3	29.535	0.001
GYAE	7	0.06	2	116.666	0.001
QDW	7	0.54	4	12.962	0.001
SIW	6	0.32	3	18.749	0.001
GVAE	6	0.27	3	21.978	0.001

Supplementary Table 4. Enriched CDR3 GLIPH motifs of TCRs from TILs of MUT2 library. P values were calculated using GLIPH package calculation.

Motif	Counts	avgRef	topRef	OvE	p-value
QDT	33	0.75	4	43.824	0.001
NER	21	0.03	1	656.249	0.001
YA	21	0.79	6	26.582	0.001
DW	21	1.64	7	12.773	0.001
YAE	21	0.09	2	233.333	0.001
QNT	19	0.83	6	22.836	0.001
AET	19	1.1	6	17.241	0.001
DWG	16	0.43	5	36.529	0.001
NSD	14	0.08	2	162.79	0.001
SQN	13	0.31	3	41.401	0.001
SNER	12	0	1	1714.285	0.001
SQNT	12	0.03	1	307.692	0.001
RDW	12	0.48	5	24.948	0.001
NQA	11	0.03	1	282.051	0.001
SAE	11	0.47	3	23.109	0.001
NQDT	10	0.01	1	666.666	0.001
SAET	10	0.06	2	158.73	0.001
NQD	10	0.04	1	232.558	0.001
RDWG	9	0.13	3	66.176	0.001
GDA	9	0.25	3	34.883	0.001
NTGQ	9	0.02	2	391.304	0.001
GQD	8	0.69	5	11.444	0.001
GQDT	8	0.17	3	46.511	0.001
NYA	7	0.02	2	291.666	0.001
NYAE	7	0.01	1	636.363	0.001
SDA	6	0.32	4	18.633	0.001
GNQA	6	0	1	1200	0.001
ANSD	5	0.01	1	416.666	0.001
QGNT	5	0.41	3	11.99	0.001
GENT	5	0.26	3	19.23	0.001
DQD	5	0.05	2	98.039	0.001
DRAN	5	0.2	3	24.271	0.001
ANS	5	0.46	4	10.822	0.001
GYAE	4	0.03	1	121.212	0.001
GGAR	4	0.21	3	18.518	0.001
SRDW	4	0.06	2	62.499	0.001
DER	4	0.06	2	57.971	0.001
SQDT	4	0.26	2	15.267	0.001
DWGG	4	0.04	1	86.956	0.001
GGSQ	4	0.15	2	25.974	0.001
DQDT	4	0	1	800	0.001
GYA	4	0.08	2	45.454	0.001
WDRG	4	0.23	3	16.877	0.001
GGRY	4	0.26	3	15.037	0.001
GNQD	4	0	1	1000	0.001
GGYA	4	0.01	1	266.666	0.001
RYA	4	0.03	1	121.212	0.001
TSAE	4	0.02	2	153.846	0.001
RYAE	4	0	1	444.444	0.001

Supplementary Table 5. List of the primers, donor oligonucleotide and gRNA sequences

Name	Sequence	Description
1036_seq_f	CAGCCATACGGAGCTTGCAGACACT	Forward primer for amplification of 1036 mutation locus
1036_seq_r	ACACGCTCTGAACCCATCCACTAGG	Reverse primer for amplification of 1036 mutation locus
1036_seq_s	CTGGATTTGAGCGAGGAGTGGGTCT	Internal sequencing primer for sequencing of 1036 mutation locus (used with amplicon from 1036_seq_f + 1036_seq_r PCR)
1036_correction_donor	CTTACCTGTGGGGGGAGCTTGTCACC GTCCAACAGCTTCACCTTGGTCTCCA ATGGCCTGATGTAAGTGGACGAGGGA TCTGAAGAACAGAGGGGAAGAAG	Donor oligonucleotide for correction of 1036 mutation to wild type
1036_regen_donor	CTTACCTGTGGGGGGAGCTTGTCACC GTCCAACAGCTTCACCTTGGTCTCAA ATGGCCTGATGTAAGTGGACGAGGGA TCTGAAGAACAGAGGGGAAGAAG	Donor oligonucleotide for reversion of wild type 1036 locus to 1036 mutation
1036_correction_sgrna	AGCTTCACCTTGGTCTCAAA	Guide RNA protospacer sequence for correction of 1036 mutation to wild type
1036_regen_sgrna	AGCTTCACCTTGGTCTCCAA	Guide RNA protospacer sequence for reversion of wild type 1036 locus to 1036 mutation

Supplemental References:

[1] Suzuki, R. and Shimodaira, H. (2006) "Pvclust: an R package for assessing the uncertainty in hierarchical clustering", Bioinformatics, 22 (12): 1540-1542.
