# Supplementary material for: Reversion analysis reveals the in vivo immunogenicity of a poorly MHC I-binding cancer neoepitope
Source: Nat Commun. 2021 Nov 5;12:6423. doi: 10.1038/s41467-021-26646-5 (PMC8571378; doi:10.1038/s41467-021-26646-5)
Supplement: Supplementary file 3 — Reporting Summary [file 41467_2021_26646_MOESM3_ESM.pdf]

nature research

Corresponding author(s): Pramod K Srivastava

Last updated by author(s): Jul 23, 2021

Reporting Summary

Nature Research wishes to improve the reproducibility of the work that we publish. This form provides structure for consistency and transparency in reporting. For further information on Nature Research policies, see our Editorial Policies and the Editorial Policy Checklist.

Statistics

For all statistical analyses, confirm that the following items are present in the figure legend, table legend, main text, or Methods section.

n/a	Confirmed	
☐	☒	The exact sample size (n) for each experimental group/condition, given as a discrete number and unit of measurement
☐	☒	A statement on whether measurements were taken from distinct samples or whether the same sample was measured repeatedly
☐	☒	The statistical test(s) used AND whether they are one- or two-sided<br>*Only common tests should be described solely by name; describe more complex techniques in the Methods section.*
☐	☒	A description of all covariates tested
☐	☒	A description of any assumptions or corrections, such as tests of normality and adjustment for multiple comparisons
☐	☒	A full description of the statistical parameters including central tendency (e.g. means) or other basic estimates (e.g. regression coefficient) AND variation (e.g. standard deviation) or associated estimates of uncertainty (e.g. confidence intervals)
☐	☒	For null hypothesis testing, the test statistic (e.g. F, t, r) with confidence intervals, effect sizes, degrees of freedom and P value noted<br>*Give P values as exact values whenever suitable.*
☒	☐	For Bayesian analysis, information on the choice of priors and Markov chain Monte Carlo settings
☒	☐	For hierarchical and complex designs, identification of the appropriate level for tests and full reporting of outcomes
☒	☐	Estimates of effect sizes (e.g. Cohen's d, Pearson's r), indicating how they were calculated

Our web collection on statistics for biologists contains articles on many of the points above.

Software and code

Policy information about availability of computer code

Data collection	Q Exactive HF and or HF-X mass spectrometer<br>SA3800 (Sony Imaging Products & Solutions Inc.)<br>built in software of HiSeq4000 system (Illumina)<br>built in software of BD LSR II-B

Data analysis	MaxQuant platform version 1.5.5.1<br>Skyline (MacCoss Lab, Skyline v19.1.0.193, Seattle, USA)<br>MSConvert (Proteowizard, Palo Alto, CA 94304, USA) release of 3.0.10577<br>pLabel (Version 2.4.0.8, pFind studio, Sci. Ac., China)<br>FCS Express 6 software (6.06.0022, De Novo Software, CA)<br>Cell Ranger Single-Cell Software Suite v.3.0 511 (10× Genomics)<br>Cell Ranger v.3.0 count pipeline<br>"cellranger 516 aggr" pipeline<br>Cell Ranger pipeline output, the 'feature (gene) vs cell' count matrix<br>SC1 tool version 1.0<br>Ward's Hierarchical Agglomerative Clustering algorithm<br>paratope hotspots (GLIPH) algorithm gliph-1.0<br>igraph R package 1.2.6<br>Rosetta and the ref2015 energy function<br>Rosetta FastRelax<br>R stats package<br>Prism 5.0 (GraphPad)

For manuscripts utilizing custom algorithms or software that are central to the research but not yet described in published literature, software must be made available to editors and reviewers. We strongly encourage code deposition in a community repository (e.g. GitHub). See the Nature Research guidelines for submitting code & software for further information.

Data

Policy information about availability of data

All manuscripts must include a data availability statement. This statement should provide the following information, where applicable:

- Accession codes, unique identifiers, or web links for publicly available datasets
- A list of figures that have associated raw data
- A description of any restrictions on data availability

Single Cell RNA-Seq and TCR-Seq data generated in this study have been deposited in the GEO database under accession code of GSE171100 https://www-ncbi-nlm-nih-gov.ezproxy.u-pec.fr/geo/query/acc.cgi?acc=GSE171100] and in the Supplemental Information Data File with this paper. There are no restrictions on data availability.

Field-specific reporting

Please select the one below that is the best fit for your research. If you are not sure, read the appropriate sections before making your selection.

☒ Life sciences ☐ Behavioural & social sciences ☐ Ecological, evolutionary & environmental sciences

For a reference copy of the document with all sections, see nature.com/documents/nr-reporting-summary-flat.pdf

Life sciences study design

All studies must disclose on these points even when the disclosure is negative.

Sample size	Sample sizes were chosen based on our previous experience with similar types of experiments as published in Ebrahimi-Nik et al. JCI Insight 2019 4, doi:10.1172/jci.insight.129152
Data exclusions	No data were excluded from analyses.
Replication	All experimental findings were reliably reproduced; the experiments were carried out between 2 and 4 times.
Randomization	All mice that participated in a study were placed in a single container, and were randomly and picked and assigned sequentially to individual groups. This applies to Figs. 1, 2 and 3 as well as Supplementary Figs. 1,2,3,4,5,6 and 8. Randomization was not relevant to any other figures or tables, since no organisms were involved.
Blinding	Blinding was not relevant since all assessments were by objective measurable criteria and no data were excluded.

Reporting for specific materials, systems and methods

We require information from authors about some types of materials, experimental systems and methods used in many studies. Here, indicate whether each material, system or method listed is relevant to your study. If you are not sure if a list item applies to your research, read the appropriate section before selecting a response.

Materials & experimental systems

n/a	Involved in the study	
☐	☒	Antibodies
☐	☒	Eukaryotic cell lines
☒	☐	Palaeontology and archaeology
☐	☒	Animals and other organisms
☒	☐	Human research participants
☒	☐	Clinical data
☒	☐	Dual use research of concern

Methods

n/a	Involved in the study	
☒	☐	ChIP-seq
☒	☐	Flow cytometry
☒	☐	MRI-based neuroimaging

Antibodies

Antibodies used

The dilutions at which the antibodies were used is described in the manuscript.
Fixable Viability Dye eFluor® 780 (65-0865-14, eBioscience)
PE anti-mouse CD45 Antibody (103106, clone 30-F11, Biolegend)
Mouse FCR blocking Reagent (130-092-575, Miltenyi Biotech)
InVivoMAb anti-CTLA4 (BE0164, Clone: 9D9, Bio X Cell)
InVivoMAb anti-CD8 (BE0061, Rat IgG2b, clone 2.43, Bio X Cell)
InVivoMAb rat IgG2b isotype control (BE0090, Rat IgG2b, clone LTF-2, Bio X Cell)
InVivoMAb anti-CD4 (BE0003-1, Rat IgG2b, clone GK1.5, Bio X Cell)
H2-Ad, anti-mouse I-Ad mAb (39-10-8, BioLegend) Catalog no 115002
mouse IgG3 isotype control (m078-3, clone 6A3, Medical & Biological Laboratories Co. Ltd.)
goat anti-mouse IgG3-PE (sc-3767, Santa Cruz Biotechnology, Inc.)
H2-E, anti-I-Ed mAb (115002, clone 14-4-4S, Thermo Fisher)
mouse IgG2a isotype control (m076-3, clone 6H3, Medical & Biological Laboratories Co. Ltd.)
goat F(ab')2 Anti-mouse Ig-PE (1012-09, Southern Biotechnology Associates Inc.)

Validation

Information re validation is provided for each reagent on the vendor's website.

The InVivoMAB Anti-CTLA4 can be used in vivo or for western blot as stated by the manufacturer.

The InVivoMAB Anti-CD8 and anti-CD4 are against Mouse CTL clones L3 and V4 and have depletion activity when used in vivo as stated by the manufacturer.

The InVivoMAB Rat IgG2b reacts with keyhole limpet hemocyanin (KLH). Because KLH is not expressed by mammals this antibody is ideal for use as an isotype-matched control for rat IgG2b antibodies in most in vivo and in vitro applications

Eukaryotic cell lines

Policy information about cell lines

Cell line source(s)

Meth A cells that have been in our lab since 1988 were originally obtained from Lloyd J Old. Meth A cells were passaged in ascites and were determined to be free from mycoplasma contamination.

Authentication

The cell lines have been authenticated by cell surface markers, morphological criteria, and their behavior upon transplantation into animals.

Mycoplasma contamination

All cell lines are periodically tested for mycoplasma contamination, and were not contaminated at last test.

Commonly misidentified lines
(See ICLAC register)

No commonly misleading cell lines were used in the study

Animals and other organisms

Policy information about studies involving animals; ARRIVE guidelines recommended for reporting animal research

Laboratory animals

The following information is provided in the manuscript.

BALB/cJ mice (6-8 week female, stock # 000651) were purchased from the Jackson Laboratory and maintained in our specific pathogen-free mouse facilities under ethical approval from the Institutional Animal Care and Use Committee of the University of Connecticut School of Medicine. Twelve light/12 dark cycle was used for mice housing. The temperature of the mice room and cages were kept around 65- 75 degrees Fahrenheit.

Wild animals

No wild animals were used in the study.

Field-collected samples

No field collected samples were used in the study.

Ethics oversight	Institutional Animal Care and Use Committee, University of Connecticut School of Medicine

Note that full information on the approval of the study protocol must also be provided in the manuscript.
